# Editorial: Cardiac rehabilitation

**DOI:** 10.3389/fresc.2026.1779058

**Published:** 2026-02-23

**Authors:** Buket Akinci, Buse Ozcan Kahraman, Melih Zeren, Svetlana Stoica

**Affiliations:** 1 Department of Physiotherapy and Rehabilitation (English), Faculty of Health Sciences, Biruni University, Istanbul, Türkiye; 2 Department of Cardiopulmonary Physiotherapy and Rehabilitation, Faculty of Physical Therapy and Rehabilitation, Dokuz Eylul University, Izmir, Türkiye; 3 Department of Physiotherapy and Rehabilitation, Faculty of Health Sciences, Bakircay University, Izmir, Türkiye; 4 Victor Babes University of Medicine and Pharmacy of Timisoara, Timisoara, Romania; 5 Institute of Cardiovascular and Heart Diseases of Timișoara, Timisoara, Romania

**Keywords:** cardiac rehabilitation, digital and hybrid rehabilitation, health equity, patient-centered care, psychosocial determinants

Cardiac rehabilitation (CR) is one of the most effective evidence-based interventions in cardiovascular medicine, consistently demonstrating benefits in mortality reduction, functional capacity, and quality of life. Yet, despite its well-established efficacy, CR remains underutilized and unevenly implemented worldwide. Traditional models often anchored in standardized exercise prescriptions and biomedical risk factor control have struggled to address the growing complexity of contemporary cardiovascular populations. Patients increasingly present with multimorbidity, psychological distress, cognitive impairment, persistent post-viral symptoms, and environmental risk exposure, all of which influence rehabilitation engagement and outcomes. The studies included in this Research Topic collectively demonstrate that cardiac rehabilitation must evolve into a multidimensional, person-centered, and adaptive system of care, capable of addressing the complex and interrelated determinants of cardiovascular health.

A central contribution of this Research Topic is the consistent emphasis on behavioral and psychosocial determinants as foundational elements of rehabilitation success. Using a health ecology framework, Li et al. show that health promotion behaviors following percutaneous coronary intervention (PCI) are shaped by interacting individual, psychological, and social factors, including health literacy, resilience, sleep quality, and perceived support. This multilevel perspective moves beyond individual responsibility and provides a conceptual basis for designing rehabilitation programs that intervene across ecological layers. Complementary findings from Guo et al. and Lu et al. further highlight the importance of fear of movement, self-efficacy, and therapeutic relationships. By demonstrating that psychologically assisted CR and theory-driven nursing interventions can significantly improve engagement and self-management, these studies reinforce the need to embed psychological care, interpersonal continuity, and behavioral theory into routine CR practice.

Equally prominent across the Research Topic is the role of context, equity, and culture in shaping rehabilitation access and effectiveness. FAITH! Cardiovascular Health and Wellness Program exemplifies how community-based participatory research can generate sustainable cardiovascular prevention strategies grounded in trust, cultural relevance, and shared ownership (Brewer et al.). Similarly, studies conducted in Romania and the Philippines illustrate both the potential and the limitations of educational and digital interventions in underrepresented or resource-constrained settings (Trani et al., Serbanoiu et al.). Serbanoiu et al. show that telemonitoring can improve engagement with blood pressure self-monitoring, while Trani et al. demonstrate that structured education can facilitate behavioral change in people living with type 2 diabetes. Together, these findings emphasize that technology and education are most effective when embedded within supportive rehabilitation ecosystems, aligned with local needs, health literacy levels, and systemic capacity.

The Research Topic also broadens the clinical scope of CR by addressing emerging and neglected patient populations. Du et al. provide important evidence that individuals with long COVID and coexisting coronary heart disease experience compounded impairments across physical, psychological, and sleep domains and that structured CR can deliver meaningful multidimensional benefits. Zhang et al. further extend rehabilitation boundaries by highlighting postoperative cognitive dysfunction (POCD) as a modifiable outcome in elderly cardiac surgery patients. Their synthesis supports a shift from reactive to proactive rehabilitation models that integrate cognitive training, exercise, and technology-based interventions across the perioperative continuum. Collectively, these studies reinforce the adaptability of CR as a platform for managing complexity, multimorbidity, and long-term functional outcomes.

Innovation in rehabilitation delivery models emerges as another unifying theme. Chait et al.’s COVID-19 natural experiment demonstrates that reduced group sizes and increased individual attention substantially improve program completion without compromising functional gains. These findings challenge efficiency-driven assumptions underlying group-based CR and highlight the importance of personalization in sustaining adherence. As hybrid and remote models become increasingly prevalent, this work underscores the need to balance scalability with individualized support.

Importantly, this Research Topic advances CR beyond traditional lifestyle-focused paradigms by incorporating environmental determinants of cardiovascular health. Supervia et al. address air pollution an established yet rarely operationalized cardiovascular risk factor in rehabilitation showing that brief, structured education can significantly enhance patients' understanding of actionable environmental health concepts. This contribution highlights an important gap in current CR curricula and suggests that environmental health literacy may represent a novel and impactful component of secondary prevention, particularly in urban and climate-affected regions.

At the mechanistic level, Zou and Hao offer a forward-looking perspective by positioning brain-derived neurotrophic factor (BDNF) as a potential mediator of exercise-induced cardiovascular benefits through a brain–heart axis. By linking neuroplasticity, exercise, and cardiac repair, this work provides a biological framework that complements the behavioral and psychosocial insights of the Research Topic. Although further validation is needed, such mechanistic perspectives open pathways toward precision rehabilitation, where interventions may be tailored to individual biological responsiveness.

Taken together, the contributions of this Research Topic support a redefinition of cardiac rehabilitation as an integrated system of care rather than a standardized intervention. Future research should prioritize longitudinal and interventional studies that test multilevel, theory-informed rehabilitation models, while incorporating psychological, cognitive, and environmental outcomes as core endpoints of cardiac rehabilitation. In parallel, there is a need to evaluate hybrid and personalized delivery models that balance adherence, equity, and scalability, alongside mechanistic research linking biological markers such as BDNF to functional and behavioral outcomes and implementation studies exploring how community-based and culturally adapted rehabilitation models can be sustainably embedded within diverse health systems.

In conclusion, this Research Topic demonstrates that advancing cardiac rehabilitation requires expanding both its scope to include psychosocial, cognitive, environmental, and biological dimensions and its depth, through personalization, contextual sensitivity, and mechanistic insight. By aligning clinical practice with the realities of modern cardiovascular health, the work presented here provides a strong foundation for the next generation of rehabilitation research and practice, aimed not only at prolonging life, but at enhancing resilience, participation, and long-term quality of life across the cardiovascular continuum.

